# Integrative Proteomics and Phosphoproteomics Profiling of Chronic Enteropathy Associated with *SLCO2A1* Gene Reveals Mucosal Barrier Impairment and Focal Adhesion Pathway Alterations

**DOI:** 10.3390/biomedicines14071412

**Published:** 2026-06-23

**Authors:** Zhixin Xie, Taotao Han, Dong Wu, Jingnan Li, Aiming Yang, Yue Li, Qiang Wang

**Affiliations:** 1 Department of Gastroenterology, Peking Union Medical College Hospital, Chinese Academy of Medical Sciences & Peking Union Medical College, Beijing 100730, China; xiezx2001@163.com (Z.X.); hantao.t@163.com (T.H.); wudong061002@aliyun.com (D.W.); pumcjnl@126.com (J.L.); yangam2020@126.com (A.Y.); 2 Eight-Year Medical Doctor Program, Chinese Academy of Medical Sciences & Peking Union Medical College, Beijing 100730, China

**Keywords:** *SLCO2A1*, chronic enteropathy, proteomics, phosphoproteomics, focal adhesion, mucosal barrier, Crohn’s disease

## Abstract

**Background**: Chronic enteropathy associated with the *SLCO2A1* gene (CEAS) is a rare disease characterized by multiple small intestinal ulcers whose pathogenesis remains poorly understood. This study aimed to characterize the proteomic and phosphoproteomic profiles of CEAS and to identify molecular pathways involved in its pathogenesis. **Methods**: Quantitative proteomics and phosphoproteomics were performed on intestinal mucosal tissues from patients with CEAS (*n* = 3), Crohn’s disease (CD, *n* = 3), and healthy controls (*n* = 3). Differentially expressed proteins (DEPs) and differentially phosphorylated proteins (DPPs) were analyzed using functional enrichment, gene set enrichment analysis (GSEA), protein–protein interaction (PPI) networks, and integrative analysis. **Results**: A total of 900 DEPs were identified in CEAS and 277 in CD relative to controls, including 717 CEAS-specific proteins. CEAS-specific alterations were strongly enriched in focal adhesion and extracellular matrix-related pathways, whereas shared proteins between CEAS and CD were primarily associated with epithelial barrier function, including tight junction and adherens junction pathways. GSEA revealed that CEAS was characterized by upregulation of tissue remodeling and focal adhesion pathways, accompanied by suppression of digestive and metabolic processes, while CD exhibited prominent adaptive immune activation. PPI network analysis identified *POSTN*, *CDH1*, *TLN1*, and *VIM* as candidate hub proteins; however, none retained significance after FDR correction, whereas brush-border components (*CDHR2*, *MUC3A*, *MUC13*, *ALPI*) and actin cytoskeletal regulators remained the most statistically robust alterations. Integrated analysis further highlighted focal adhesion-related proteins with coordinated expression and phosphorylation changes. **Conclusions**: This exploratory study provides the first integrative proteomic and phosphoproteomic characterization of CEAS, suggesting that impairment of the intestinal brush border and mucosal barrier, together with actin cytoskeletal reorganization, may distinguish CEAS from immune-dominant CD. These findings are hypothesis-generating and require validation in larger cohorts.

## 1. Introduction

Chronic enteropathy associated with the *SLCO2A1* gene (CEAS) is a rare gastroenterological disease characterized by multiple ulcers in the small intestine, accompanied by chronic bleeding and protein loss. While nearly all segments of the stomach and small intestine can be affected, involvement of the ileum, except for the terminal segment, is most frequently observed [1,2]. Without genetic testing, the lack of histological specificity makes it difficult to distinguish CEAS from other gastrointestinal ulcerative diseases, such as Crohn’s disease (CD) and cryptogenic multifocal ulcerous stenosing enteritis (CMUSE) [2]. *SLCO2A1* encodes OATP2A1, which is a member of the solute carrier organic anion transporter family and plays an integral part in prostaglandin E (PGE) transportation. Previously, researchers hypothesized that the elevated PGE_2_ levels resulting from OATP2A1 dysfunction were the primary cause of CEAS [3,4,5]. However, the limited therapeutic efficacy of COX-2 inhibitors, which lower PGE_2_ levels, suggests that additional pathogenic mechanisms may contribute to CEAS [6]. Proteomics is a powerful approach for analyzing and defining protein expression patterns, making it suitable for the exploration of unrecognized proteins related to specific diseases. Therefore, understanding the characteristics of CEAS protein expression profiles will aid in identifying the underlying etiology.

This study aimed to comprehensively characterize the proteomic and phosphoproteomic profiles of CEAS and to elucidate potential molecular pathways involved in its pathogenesis. To delineate the specific features of CEAS, we selected control groups comprising individuals with CD and healthy individuals without intestinal diseases as CD represents a major differential diagnosis for CEAS given the significant overlap in their clinical presentations. Our findings provide candidate loci that may contribute to the pathogenesis of CEAS and lay the groundwork for future research.

## 2. Materials and Methods

### 2.1. Sample Collection and Preparation

Small intestinal mucosal tissues were obtained from three CEAS patients, three CD patients (lesioned mucosa), and three individuals with relatively healthy mucosa at Peking Union Medical College Hospital (PUMCH). Healthy control specimens were obtained from patients who underwent small bowel resection for intestinal ischemia caused by trauma. Tissue was sampled from macroscopically and histologically normal mucosa distant from the ischemic segment, confirmed by routine pathological examination to be free of inflammatory, neoplastic, or other mucosal abnormalities. Control subjects had no clinical history of chronic gastrointestinal disease. Detailed clinical and sample characteristics of all participants are provided in Supplementary Table S3. All specimens were immediately fixed in 10% neutral buffered formalin and paraffin-embedded for preservation. The study utilized residual archival tissue paraffin blocks obtained after routine pathological diagnosis. All samples were anonymized before analysis. Formalin-fixed paraffin-embedded (FFPE) tissue sections were deparaffinized using xylene (56 °C, 2 × 60 min) followed by graded ethanol washes (100% and 95%) and water rinses. Samples were homogenized using steel beads. Proteins were extracted using the Fast Proteomics Sample Preparation Kit (PF-226089; Biomsomics, Beijing, China) with 3 h incubation at 100 °C and quantified via BCA assay (Thermo Fisher Scientific, Waltham, MA, USA) with a 0–2 μg/μL BSA standard curve.

### 2.2. Protein Digestion and LC-MS/MS

For global proteomics, proteins were reduced (25 mM DTT, 37 °C, 1 h), alkylated (50 mM IAA, room temperature, 30 min), and digested overnight with trypsin (1:50 *w*/*w*) using 10 kDa centrifugal filters. For phosphoproteomics, peptide samples were enriched using High-Select™ TiO_2_ Phosphopeptide Enrichment Kit (Thermo Fisher Scientific, A32993). Peptides were separated on a nanoViper C18 column (75 μm × 250 mm) with a 90 min (proteomics) or 60 min (phosphoproteomics) gradient (1–35% solvent B; solvent A, 0.1% FA in water; solvent B, 80% ACN/0.1% FA) at 1.2 μL/min. Proteomic data were acquired on an Orbitrap Exploris 480 in data-independent acquisition (DIA) mode (MS1 resolution 120,000, MS2 resolution 30,000; scan range 350–1200 *m*/*z*; stepped HCD 25/30/35%; 80 DIA windows). Phosphoproteomic data were acquired on a Q-Exactive HF in data-dependent acquisition (DDA) mode (MS1 resolution 120,000, MS2 resolution 15,000; scan range 350–1500 *m*/*z*; HCD 30%; dynamic exclusion 30 s).

### 2.3. Data Processing

Proteomic raw data were analyzed with DIA-NN 1.8.11 and phosphoproteomic raw data with MaxQuant 2.0.1.0, both searched against the UniProt human database (uniprot_human, 81,803 sequences, 27 March 2023 release), allowing up to two missed cleavages, a precursor and fragment mass tolerance of 10 ppm and 0.02 Da, a fixed modification of carbamidomethyl (C), and variable modifications of methionine oxidation and N-terminal acetylation; phospho (STY) was included as an additional variable modification for the phosphoproteomic search. Both searches applied a 1% false discovery rate (FDR) at the peptide-spectrum match (PSM) and protein levels and required at least one protein-specific peptide. Quantitative intensities were normalized and log2-transformed in Perseus (v1.5.5.1) prior to statistical analysis. For the proteomic dataset, 4753 proteins were quantified and retained, defined as proteins with at least two valid (non-zero) values in each group; no imputation of missing values was performed. For the phosphoproteomic dataset, 3250 phosphosites were identified, of which 2079 were class I sites (localization probability > 0.75); 921 sites with at least two valid values per group were retained for quantitative comparison. As the proteomic (DIA) and phosphoproteomic (DDA) datasets were processed independently, phosphosite intensities were not normalized to total protein abundance, and phosphorylation changes should therefore be interpreted alongside total protein-level alterations where relevant. All nine samples were prepared and acquired in a single batch under identical protocols; no formal batch-effect correction was applied. Quality-control metrics, including mass accuracy, inter-sample correlation, principal component analysis, and pre-/post-normalization intensity distributions, are provided in Supplementary Figure S3.

Differentially expressed proteins (DEPs) and differentially phosphorylated sites were defined as those with a fold change ≥ 1.5 or ≤0.67 and a nominal *p* value ≤ 0.05 (Student’s *t*-test). Gene-level differentially phosphorylated proteins (DPPs) were obtained by deduplication of significant sites. Given the limited sample size and the exploratory nature of this study, nominal significance thresholds were used for the primary analyses to maximize the capture of candidate findings. To assess robustness under stringent multiple-testing correction, Benjamini–Hochberg (BH) FDR correction was additionally applied to the original *p*-values across the full quantified proteome (4753 proteins) and phosphosite matrix (921 sites) for all three comparisons as a sensitivity analysis (Supplementary Table S2).

### 2.4. Bioinformatic Analyses

DEPs were categorized by Venn diagram analysis into three groups: (i) CEAS-specific DEPs, identified in CEAS vs. Normal but not in CD vs. Normal; (ii) shared DEPs, present in both CEAS vs. Normal and CD vs. Normal; and (iii) CEAS–CD discriminating DEPs, differentially expressed in all three comparisons (CEAS vs. Normal, CD vs. Normal, and CEAS vs. CD), representing proteins that are altered in both diseases relative to controls but also differ significantly between CEAS and CD. The same classification was applied to differentially phosphorylated proteins. As the primary aim of this study was to characterize the molecular features of CEAS, with CD serving as a clinically overlapping comparator rather than an independent disease of investigation, DEPs and DPPs uniquely altered in CD (present in CD vs. Normal but absent in CEAS vs. Normal) were not subjected to further downstream analysis; their complete lists are nonetheless provided in the Supplementary Materials. Volcano plots were generated using the ggVolcano R package. Hierarchical clustering and heatmap visualization were performed using the pheatmap package in R on the top 100 most variable proteins with Z-score normalization; the gene dendrogram was cut into 5 clusters using the cutree function, and Gene Ontology (GO) biological process enrichment was performed for each cluster using the clusterProfiler package (*p* < 0.05). GO enrichment analysis encompassing biological process (BP), cellular component (CC), and molecular function (MF) categories was conducted using DAVID 2021 (https://davidbioinformatics.nih.gov, accessed on 10 June 2026). Kyoto Encyclopedia of Genes and Genomes (KEGG) pathway enrichment was performed using the hypergeometric test (*p* < 0.05).

Gene set enrichment analysis (GSEA) was performed using the *clusterProfiler* R package to detect coordinated pathway-level expression changes. For each comparison, all quantified proteins were ranked by log_2_ (Ratio). GSEA was conducted with the *gseGO* and *gseKEGG* functions (gene set size: 10–500). Significance criteria were |NES| > 1, nominal *p* < 0.05, and FDR < 0.25. GSEA was applied to both proteomic and phosphoproteomic data for all three comparisons. To evaluate whether the principal functional themes were preserved under stringent statistical correction, GO over-representation analysis (enrichGO, clusterProfiler) with BH correction and the full quantified proteome as background was additionally performed on the subset of proteins retaining significance after FDR correction in the CEAS vs. Normal comparison.

Protein–protein interaction (PPI) networks were constructed using the STRING database (version 12.0; confidence ≥ 0.400). Hub genes were identified using the cytoHubba plugin (Maximal Clique Centrality algorithm) in Cytoscape (v3.10.0), and the top 20 genes ranked by MCC score were extracted for each network.

### 2.5. Molecular Docking

Given the long-standing hypothesis that PGE_2_ accumulation contributes to CEAS pathogenesis, a hypothesis-driven molecular docking analysis was performed to test whether PGE_2_ might directly interact, in a non-canonical structural manner, with the four candidate hub proteins (*POSTN*, *CDH1*, *TLN1*, and *VIM*), using AutoDock 4.2 [7]. This analysis was not intended to propose these proteins as PGE_2_ receptors as PGE_2_ signaling is normally mediated by prostaglandin EP1–EP4 receptors. Three-dimensional structures of the target proteins were obtained from the Protein Data Bank: *POSTN* (PDB ID: 5YJH), *CDH1* (PDB ID: 2O72), *TLN1* (PDB ID: 6R9T), and *VIM* (PDB ID: 1GK4). The structure of PGE_2_ was retrieved from the PubChem database. Receptor and ligand files were prepared using AutoDockTools 1.5.7, including addition of polar hydrogens, assignment of Gasteiger charges, and definition of rotatable bonds (15 active torsions for PGE_2_). Docking was performed using the Lamarckian genetic algorithm with the following parameters: 50 independent GA runs, population size of 150, maximum number of energy evaluations of 2.5 × 10^6^, maximum number of generations of 27,000, mutation rate of 0.02, and crossover rate of 0.8. The docking results were clustered using a 2.0 Å RMSD tolerance and ranked by estimated free energy of binding.

## 3. Results

### 3.1. Identification and Classification of Differentially Expressed and Phosphorylated Proteins

**Figure 1:**
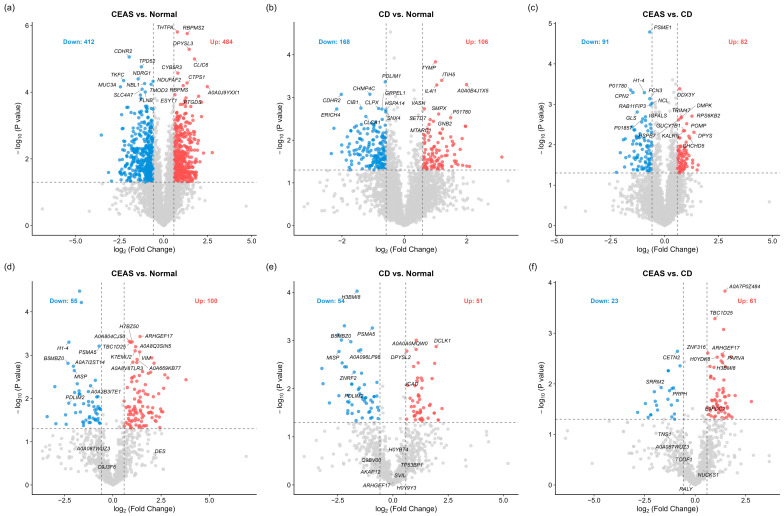
Volcano plots of differentially expressed and phosphorylated proteins. (**a**–**c**) DEPs in CEAS vs. Normal, CD vs. Normal, and CEAS vs. CD. (**d**–**f**) DPPs in the same comparisons. Red dots indicate upregulated and blue dots indicate downregulated proteins (|log_2_ FC| > 0.585 and nominal *p* < 0.05). The top 10 most significant genes in each direction are labeled.

**Figure 2:**
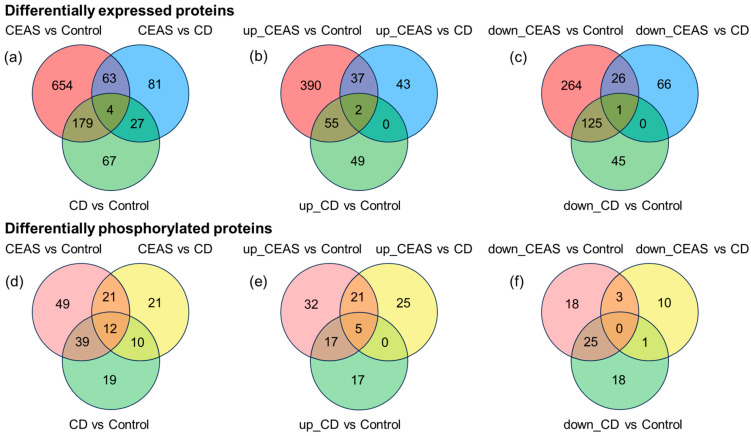
Venn diagram analysis of differentially expressed and phosphorylated proteins. (**a**) Three-way Venn diagram of all DEPs across CEAS vs. Control, CEAS vs. CD, and CD vs. Control comparisons. (**b**) Venn diagram of upregulated DEPs. (**c**) Venn diagram of downregulated DEPs. (**d**) Three-way Venn diagram of all DPPs across the same comparisons. (**e**) Venn diagram of upregulated DPPs. (**f**) Venn diagram of downregulated DPPs.

Compared with healthy controls, 900 DEPs were identified in the CEAS group (484 upregulated, 416 downregulated), and 277 DEPs in the CD group (106 upregulated, 171 downregulated). Direct comparison between CEAS and CD revealed 175 DEPs (82 upregulated, 93 downregulated in CEAS). Volcano plots demonstrated distinct expression patterns across groups (Figure 1a–c). Through Venn diagram analysis (Figure 2a–c), we identified 717 CEAS-specific DEPs, with 183 DEPs shared between CEAS and CD. Among the shared DEPs, four were also differentially expressed between CEAS and CD, including two downregulated proteins (*GRPEL1* and *HNF4A*) and two upregulated proteins (*GUCY1A1* and *THY1*), which may serve as candidate biomarkers for differentiating CEAS from CD (Table 1).

After gene-level deduplication, 121 differentially phosphorylated proteins (DPPs) were identified in CEAS vs. Normal, 80 in CD vs. Normal, and 64 in CEAS vs. CD. At the phosphorylation site level, 155 differentially phosphorylated sites were identified in CEAS vs. Normal (100 upregulated, 55 downregulated), 105 in CD vs. Normal (51 upregulated, 54 downregulated), and 84 in CEAS vs. CD (61 upregulated, 23 downregulated), with the top 10 most significant genes in each direction labeled on volcano plots (Figure 1d–f). Venn diagram analysis identified 70 CEAS-specific DPPs, 51 shared DPPs, and 12 discriminating DPPs (Table 1, Figure 2d–f).

### 3.2. Hierarchical Clustering Analysis

**Figure 3:**
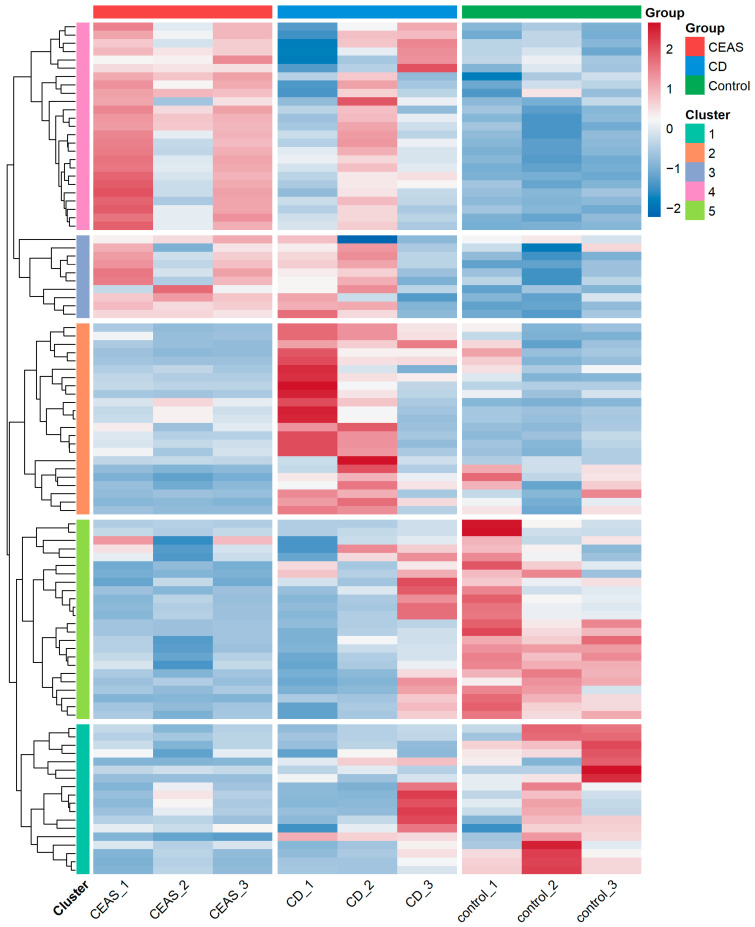
Hierarchical clustering heatmap of the top 100 most variable proteins across CEAS, CD, and Control groups. Rows represent genes (Z-score normalized) and columns represent samples. Five distinct gene clusters are indicated.

Hierarchical clustering of the top 100 most variable proteins revealed five distinct clusters with characteristic expression patterns (Figure 3, Table 2). Cluster 1 (18 genes, high in CEAS) was enriched in hydrogen peroxide catabolism and cellular oxidant detoxification (*p* = 5.20 × 10^−11^; key genes: *HBB*, *HBA1*, *HBD*, *MPO*, and *S100A9*). Cluster 2 (23 genes, low in CEAS) was associated with immunoglobulin-mediated immune response (*p* = 1.65 × 10^−9^; *IGHA1*, *IGKC*, *IGHG1*). Cluster 3 (10 genes, high in CD) was enriched in platelet aggregation and elastic fiber assembly (*MYL9*, *MYH11*, and *VCL*). Cluster 4 (25 genes, high in controls) reflected glycolytic processes (*GAPDH*, *ENO1*, *ALDOA*, and *PKM*). Cluster 5 (24 genes, heterogeneous) was linked to epithelial cell apoptosis and fibrinolysis (*FGA*, *FGB*, *FGG*, and *KRT8*). These results suggest that CEAS is characterized by enhanced oxidative stress responses and detoxification mechanisms, while CD shows distinct features related to immune activation and platelet function. Both diseases exhibited reduced glycolytic metabolism compared to healthy controls.

### 3.3. Functional Enrichment Analysis

#### 3.3.1. Proteomic Enrichment

**Figure 4:**
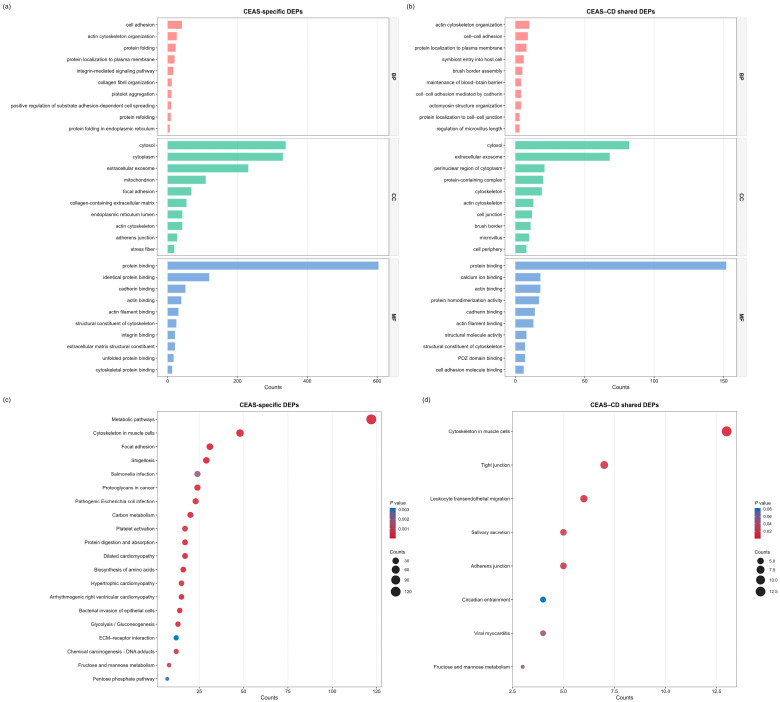
DAVID functional enrichment analysis of differentially expressed proteins. (**a**) GO enrichment of CEAS-specific DEPs. (**b**) GO enrichment of CEAS–CD shared DEPs. (**c**) KEGG pathway enrichment of CEAS-specific DEPs. (**d**) KEGG pathway enrichment of CEAS–CD shared DEPs.

GO and KEGG enrichment analyses were performed on CEAS-specific and CEAS–CD shared DEPs using DAVID (Figure 4). CEAS-specific DEPs were significantly enriched in focal adhesion (GO CC, *p* = 1.77 × 10^−20^, fold enrichment = 4.01), cytosol (*p* = 4.81 × 10^−19^), and extracellular exosomes (*p* = 2.58 × 10^−18^). GO BP analysis demonstrated enrichment in protein folding (*p* = 2.03 × 10^−7^), actin cytoskeleton organization (*p* = 2.79 × 10^−7^), and platelet aggregation (*p* = 5.65 × 10^−6^). Molecular function enrichment highlighted cadherin binding (*p* = 1.24 × 10^−12^) and actin binding (*p* = 8.62 × 10^−10^). KEGG analysis identified cytoskeleton in muscle cells as the most significantly enriched pathway (*p* = 5.86 × 10^−18^, 48 genes), followed by metabolic pathways (122 genes), and focal adhesion (*p* = 2.43 × 10^−8^, 31 genes) (Figure 4a,c). Notably, focal adhesion ranked among the top terms in both KEGG and GO analyses, suggesting its critical role in the pathogenesis of CEAS.

CEAS–CD shared DEPs exhibited functional enrichment centered on epithelial barrier integrity. GO BP analysis revealed striking enrichment in brush border assembly (*p* = 3.64 × 10^−7^, fold enrichment = 72.03), actin cytoskeleton organization, and cell–cell adhesion. These proteins were predominantly localized to the brush border (*p* = 1.51 × 10^−10^, fold enrichment = 15.30), apical plasma membrane, and microvilli. KEGG analysis demonstrated significant enrichment in the cytoskeleton in muscle cells, leukocyte transendothelial migration, tight junction, and adherens junction (Figure 4b,d). Only four DEPs discriminated CEAS from CD, precluding robust pathway enrichment.

#### 3.3.2. Phosphoproteomic Enrichment

**Figure 5:**
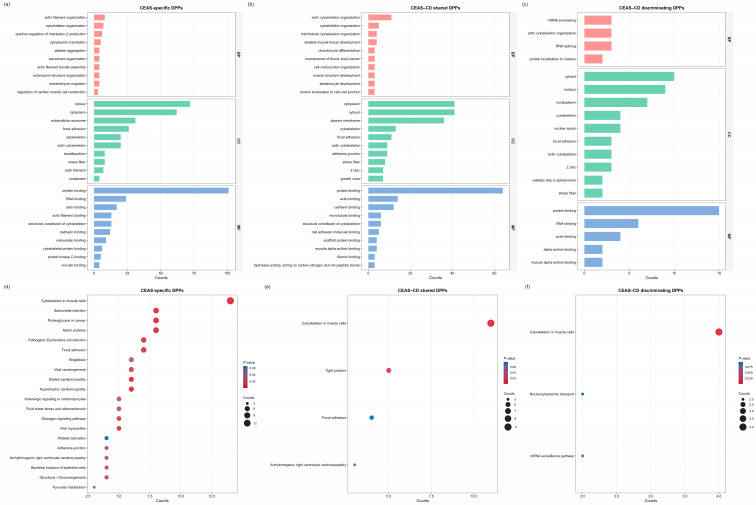
DAVID functional enrichment analysis of differentially phosphorylated proteins. (**a**) GO enrichment of CEAS-specific DPPs. (**b**) GO enrichment of CEAS–CD shared DPPs. (**c**) GO enrichment of CEAS–CD discriminating DPPs. (**d**) KEGG pathway enrichment of CEAS-specific DPPs. (**e**) KEGG pathway enrichment of CEAS–CD shared DPPs. (**f**) KEGG pathway enrichment of CEAS–CD discriminating DPPs.

Phosphoproteomic analysis of CEAS-specific alterations uncovered prominent dysregulation of cytoskeletal signaling and inflammatory pathways (Figure 5a,d). GO BP analysis revealed significant involvement in mesenchyme migration (*p* = 1.55 × 10^−6^), positive regulation of interleukin-2 production (*p* = 1.65 × 10^−6^), and actin filament organization. Differentially phosphorylated proteins localized predominantly to focal adhesions (*p* = 2.18 × 10^−13^), stress fibers, and Z discs. KEGG pathway analysis revealed significant enrichment in cytoskeleton in muscle cells (*p* = 9.94 × 10^−9^, 14 genes), motor proteins, and proteoglycans in cancer.

CEAS–CD shared DPPs showed enrichment in sarcomeric and contractile machinery, including supramolecular fiber organization, skeletal muscle thin filament assembly, and sarcomere organization. KEGG analysis highlighted cytoskeleton in muscle cells (*p* = 1.25 × 10^−7^, 11 genes) and the tight junction (Figure 5b,e). The 12 DPPs discriminating CEAS from CD were enriched in RNA splicing (*p* = 0.012), actin cytoskeleton organization (*p* = 0.013), and nuclear speckle localization, with *SYNPO2*, *LMNA*, *LMOD1*, and *PDLIM4* as core discriminating phosphoproteins (Figure 5c,f).

### 3.4. Gene Set Enrichment Analysis

**Figure 6:**
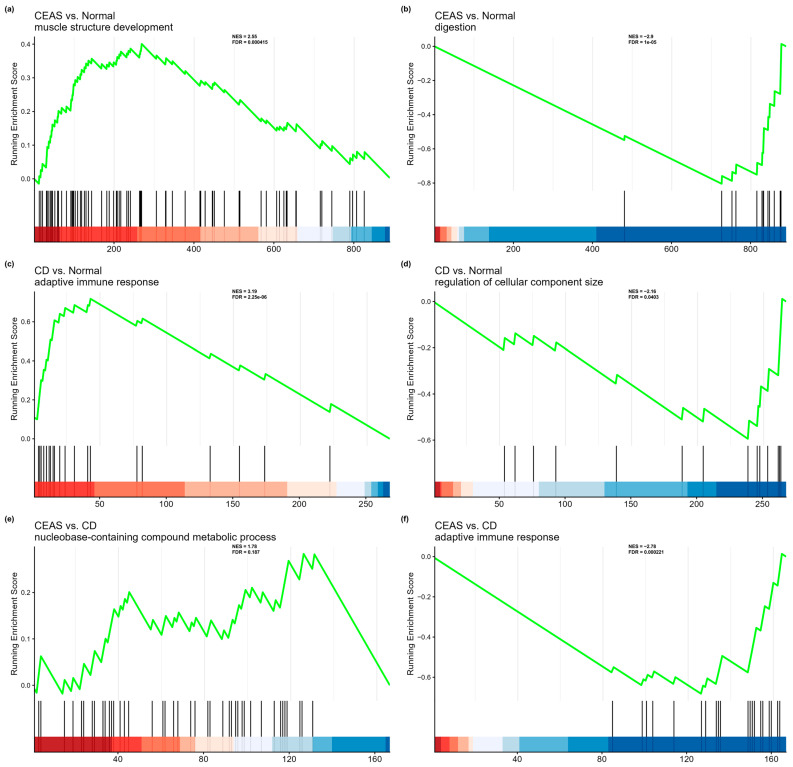
Gene set enrichment analysis (GSEA) of proteomic data. Representative enrichment plots showing the top upregulated and downregulated GO biological process pathways for each comparison. (**a**,**b**) CEAS vs. Normal. (**c**,**d**) CD vs. Normal. (**e**,**f**) CEAS vs. CD. NES and FDR values are shown within each panel. Significance criteria: |NES| > 1, nominal *p* < 0.05, and FDR < 0.25.

GSEA was performed on the full ranked proteome to detect coordinated pathway-level expression changes (Table 3, Figure 6, Supplementary Figure S1). The CEAS vs. Normal comparison yielded the largest number of significantly enriched gene sets (145 GO BP, 20 KEGG), consistent with the substantial proteomic alterations observed. Among the upregulated pathways, focal adhesion was the most significantly enriched KEGG pathway (NES = 2.22, FDR = 0.011), followed by cytoskeleton in muscle cells (NES = 2.02), integrin signaling (NES = 2.08), and the MAPK signaling pathway (NES = 1.90). Additional upregulated GO BP processes included extracellular matrix organization, collagen fibril organization, cell–matrix adhesion, and angiogenesis, indicating broad activation of tissue remodeling programs. Among the downregulated pathways, digestion (NES = −2.94) and digestive system process (NES = −2.85) were the most significantly suppressed, accompanied by lipid catabolic process, organic anion transport, and xenobiotic metabolism, reflecting a widespread loss of epithelial metabolic capacity (Figure 6a,b).

In contrast, CD vs. Normal was characterized primarily by adaptive immune response activation (NES = 3.24, FDR = 7.92 × 10^−7^), followed by immunoglobulin-mediated immune response (NES = 2.74) and B cell-mediated immunity (NES = 2.74). Only 2 KEGG pathways met significance (Figure 6c,d). Direct CEAS–CD comparison confirmed that immune activation processes were significantly more enriched in CD than in CEAS (adaptive immune response NES = −2.78 relative to CEAS), highlighting a fundamental biological distinction between these two enteropathies (Figure 6e,f). For the phosphoproteomic data, no gene sets met significance criteria in the CEAS vs. Normal comparison or in any KEGG analysis. However, in the CD vs. Normal comparison, 4 GO BP gene sets reached borderline significance (FDR ≈ 0.25), involving cellular localization and regulation of cellular process. Of note, in the CEAS vs. CD comparison, 9 GO BP gene sets were significantly enriched (FDR = 0.067–0.146), all with negative NES values, predominantly involving nucleic acid and RNA metabolic processes (top pathway: nucleic acid metabolic process, NES = −2.15, FDR = 0.067). This suggests that RNA processing and nucleic acid metabolism-related phosphorylation signaling may be more active in CD than in CEAS, complementing the phosphoproteomic DAVID enrichment finding of RNA splicing among the DPPs discriminating CEAS from CD.

### 3.5. Robustness Assessment by FDR Correction

To evaluate the statistical robustness of the differential expression findings under stringent multiple-testing correction, we applied Benjamini–Hochberg (BH) FDR correction to the original *p*-values across the full quantified proteome (4753 proteins) and phosphosite matrix (921 sites) for all three comparisons (Supplementary Table S2). In the CEAS vs. Control comparison, 128 proteins retained significance at *q* < 0.05 together with the fold-change criterion (66 upregulated, 62 downregulated), with the smallest adjusted *p*-value reaching *q* = 0.0042. In contrast, no proteins reached *q* < 0.05 in the CD vs. Control or CEAS vs. CD comparisons, and only two phosphosites reached *q* < 0.05 (both in the CEAS vs. Control comparison). This pattern is consistent with the limited statistical power inherent to a sample size of three per group and indicates that the most robust molecular signal resides in the CEAS vs. Control contrast.

Among the 128 FDR-robust proteins, several key barrier- and epithelial-function-related molecules retained strong individual significance, including the intermicrovillar adhesion protein *CDHR2* (*q* = 0.0096), the brush-border mucins *MUC3A* (*q* = 0.021) and *MUC13* (*q* = 0.045), intestinal alkaline phosphatase *ALPI* (*q* = 0.037), and the epithelial differentiation regulator *NDRG1* (*q* = 0.021), all downregulated in CEAS. These findings indicate that loss of brush-border and apical-membrane components is supported at the level of individual, statistically robust proteins.

At the pathway level, GO over-representation analysis of the 128 FDR-robust proteins (enrichGO, BH correction, and whole-proteome background) identified significant enrichment only in actin cytoskeletal cellular components, including stress fiber, contractile actin filament bundle, and actomyosin (adjusted *p* = 0.018), driven by *TPM1*, *FLNB*, *TPM3*, *ZYX*, *FSCN1*, *SORBS1*, and *DES*. No GO biological process, molecular function, or KEGG pathway survived multiple-testing correction within this restricted gene set, reflecting the reduced power of pathway-level enrichment when applied to a small, stringently filtered protein list.

Taken together, the FDR-robust subset confirms that actin cytoskeletal reorganization and the loss of individual brush-border/apical-membrane proteins represent the most statistically durable molecular features of CEAS. By contrast, the PPI-derived hub proteins (*POSTN*, *CDH1*, *TLN1*, and *VIM*) did not retain FDR-level significance (Table 4) and are interpreted as candidate findings requiring independent validation.

### 3.6. PPI Network and Hub Gene Analysis

**Figure 7:**
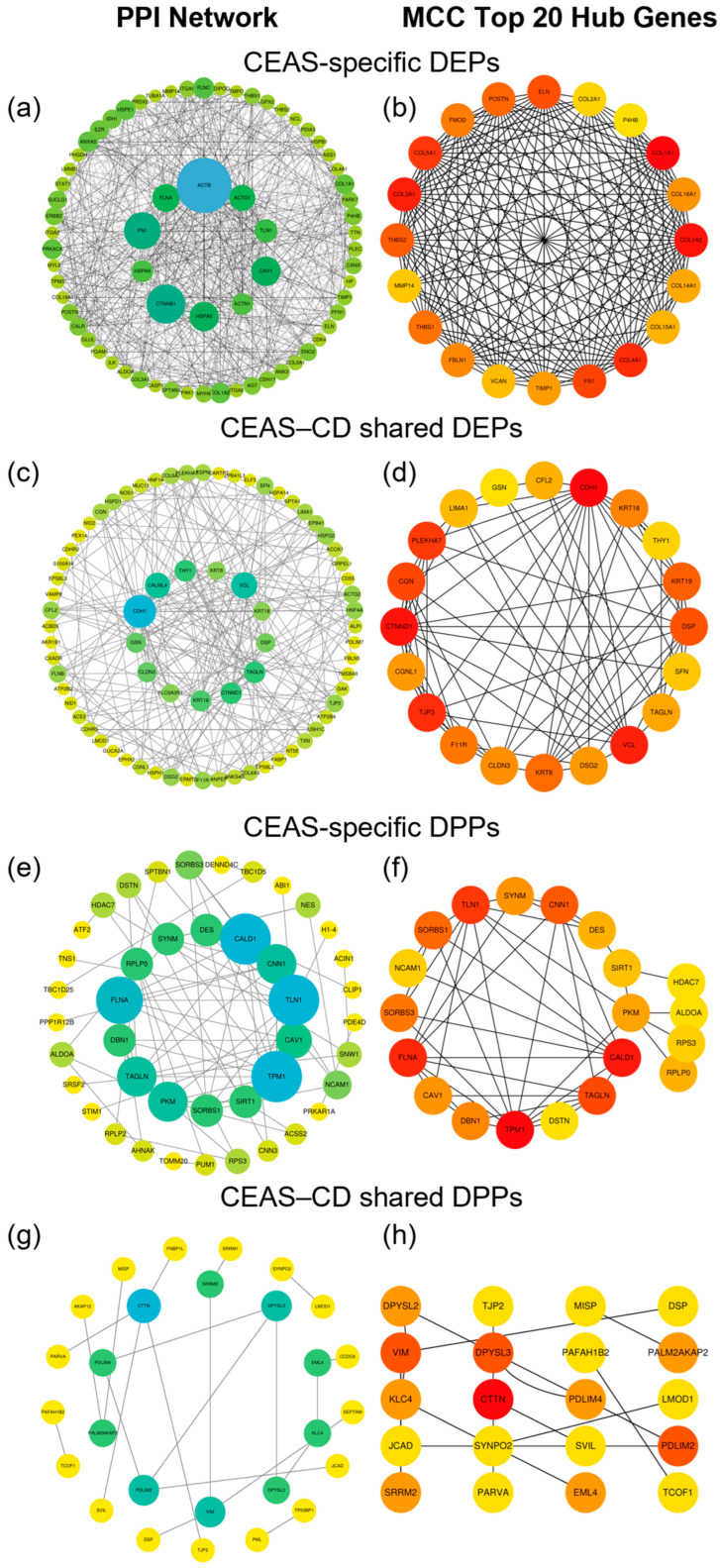
Protein–protein interaction (PPI) network and hub gene analysis. Left panels show the full STRING PPI networks; right panels show the top 20 hub genes identified by the MCC algorithm in cytoHubba. (**a**,**b**) CEAS-specific DEPs. (**c**,**d**) CEAS–CD shared DEPs. (**e**,**f**) CEAS-specific DPPs. (**g**,**h**) CEAS–CD shared DPPs. Node size and color in the network panels (**left**) scale with degree (larger/blue = more interactions, smaller/yellow = fewer); in the hub gene panels (**right**), color reflects cytoHubba MCC rank (red = highest, yellow = lowest among the top 20).

Four PPI networks were constructed to explore the functional interactions among differentially expressed and phosphorylated proteins (Figure 7, Table 4). The CEAS-specific proteomics network comprised 648 nodes. CytoHubba analysis identified collagen family members (*COL1A1*, *COL1A2*, *COL3A1*, *COL4A1*, and *COL5A1*) and ECM-associated proteins (*FN1*, *ELN*, *THBS2*, *POSTN*, and *THBS1*) as the top 10 hub genes. Additional highly connected nodes included *TIMP1*, *MMP14*, and *VCAN*, reinforcing the centrality of ECM remodeling in CEAS-specific pathogenesis.

The CEAS–CD shared proteomics network (134 nodes) was dominated by cell junction proteins. The top hub genes, including *CDH1* (E-cadherin), *CTNND1*, *VCL* (vinculin), *TJP3*, *PLEKHA7*, *CGN* (cingulin), *DSP* (desmoplakin), *KRT19*, *KRT8*, and *F11R* (JAM-A), are predominantly involved in cell–cell adhesion, tight junction assembly, and epithelial barrier integrity, suggesting that disruption of intestinal epithelial barrier function is a shared pathological mechanism between CEAS and CD.

The CEAS-specific phosphoproteomics network (43 nodes) highlighted cytoskeletal regulators (*TPM1*, *CALD1*, *FLNA*, *TLN1*, *TAGLN*, and *CNN1*) as top hubs. The identification of *TLN1* as a hub gene is particularly noteworthy as it was also identified as a key focal adhesion molecule in the integrated proteomics–phosphoproteomics analysis. The shared phosphoproteomics network (27 nodes) was led by *CTTN* (cortactin) and *VIM* (vimentin), along with multiple PDZ-LIM domain proteins (*PDLIM2* and *PDLIM4*), indicating that cytoskeletal reorganization and signal transduction at the cell cortex are shared phosphorylation-regulated processes in both diseases.

Given the central role of PGE_2_ transport dysfunction in CEAS pathogenesis, we further investigated whether PGE_2_ may directly interact with these hub proteins using molecular docking. All four proteins exhibited weak binding affinities with PGE_2_ (best ΔG ranging from −2.04 to −2.95 kcal/mol) and highly dispersed conformational clustering (46–49 clusters from 50 runs), suggesting the absence of direct PGE_2_–protein binding (Supplementary Figure S2, Supplementary Table S1).

### 3.7. Integrated Analysis of Proteomics and Phosphoproteomics

To identify key regulatory proteins, we integrated the proteomic and phosphoproteomic datasets by identifying proteins that were differentially expressed at both the total protein and phosphorylation levels. For CEAS-specific proteins, intersection analysis revealed 10 proteins altered at both levels: *EIF3J*, *TLN1*, *PHLDB1*, *GREM1*, *SYNM*, *BAD*, *FLNA*, *DES*, *SORBS1*, and *SPEG*. These proteins may represent key regulators specific to CEAS pathogenesis. For overlapping proteins between CEAS and CD, 7 proteins showed changes at both levels: *ANKS4B*, *MYH14*, *MISP*, *CGN*, *DSP*, *TJP3*, and *CXADR*. These proteins likely represent common pathogenic mechanisms shared between CEAS and CD. No overlapping proteins were found among those discriminating CEAS from CD, suggesting that the distinguishing features of these diseases may operate through distinct regulatory mechanisms at the protein expression versus phosphorylation levels.

## 4. Discussion

Integrative proteomic and phosphoproteomic profiling can provide insights into complex diseases such as CEAS, whose pathogenesis remains poorly understood. In this exploratory study, we characterized the proteomic and phosphoproteomic landscapes of CEAS and identified candidate proteins and pathways potentially involved in disease pathogenesis. To delineate the molecular features specific to CEAS, we included both patients with CD and healthy controls. CD was selected because it represents one of the most important differential diagnoses of CEAS owing to their substantial clinical and endoscopic overlap [2].

Hierarchical clustering analysis showed that the top 100 most variable proteins segregated into five functionally distinct clusters, highlighting distinct proteomic patterns across the three groups. Notably, Cluster 1, which showed high expression exclusively in CEAS, was dominated by hemoglobin subunits (*HBB*, *HBA1*, and *HBD*) and inflammatory mediators (*MPO* and *S100A9*), enriched in hydrogen peroxide catabolism and oxidant detoxification. This pattern may reflect chronic mucosal bleeding and the associated oxidative stress response resulting from persistent ulcerative injury [1,2]. In contrast, Cluster 2 (low in CEAS, variable in CD) was enriched in immunoglobulin-mediated immunity, consistent with the more prominent adaptive immune activation observed in CD [8]. The downregulation of glycolytic enzymes (Cluster 4) in both disease groups compared to controls may reflect a shared metabolic impairment of intestinal epithelial cells.

DAVID enrichment analysis further characterized the functional landscape of differentially expressed proteins. Among the most striking findings, CEAS–CD shared DEPs exhibited an exceptionally high fold enrichment in brush border assembly, alongside enrichment in actin cytoskeleton organization and cell–cell adhesion. These results indicate that disruption of the intestinal brush border, a hallmark of epithelial barrier dysfunction [9], is a convergent pathological feature of both CEAS and CD. For CEAS-specific DEPs, focal adhesion emerged as a prominent term in both GO cellular component and KEGG pathway analyses.

GSEA further revealed coordinated pathway-level alterations. GSEA distinguished CEAS from CD at the pathway level: CEAS was characterized by coordinated upregulation of tissue-remodeling programs, including extracellular matrix organization, focal adhesion, and angiogenesis, accompanied by coordinated downregulation of digestive function and lipid metabolism. In contrast, CD was defined primarily by strong upregulation of adaptive immune response and B cell-mediated immunity [8], aligning with the immunoglobulin-dominated Cluster 2 observed in our hierarchical clustering analysis. These pathway-level distinctions suggest divergent biological programs underlying these clinically overlapping enteropathies and may provide a basis for future biomarker studies aimed at improving differential diagnosis. Although impaired prostaglandin transport and elevated PGE2 levels are considered central to CEAS pathogenesis, these mechanisms alone may not fully explain the widespread proteomic alterations observed in the present study [6]. It is worth noting that, whereas GSEA of the proteomic data revealed coordinated pathway-level shifts, the phosphoproteomic data yielded few significant gene sets by GSEA. This observation is not unexpected because GSEA detects concerted, pathway-wide intensity changes, whereas phosphorylation typically regulates discrete signaling events at individual sites. The phosphoproteomic alterations reported here are therefore best interpreted as candidate site-level signaling events rather than coordinated pathway-level changes.

Through PPI network analysis, we identified several hub proteins of interest, including *POSTN*, collagen family members (*COL1A1* and *COL1A2*), and ECM-associated proteins (*FN1* and *THBS1*) in the CEAS-specific network, and *TLN1* (talin-1) in the phosphoproteomics network. Notably, none of these hub proteins retained significance after FDR correction (Table 4); as hub status reflects network topology rather than statistical robustness, we interpret them as candidates rather than established drivers. *POSTN* (periostin) has been implicated in tissue remodeling and fibrosis in a variety of chronic inflammatory and fibrotic disorders [10,11]. Talin-1 connects integrins to the actin cytoskeleton at focal adhesions [12]. Dysregulation of talin-1 has been reported to promote intestinal inflammation via the TLR/NF-κB pathway and disrupt tight-junction integrity [13,14], making its altered phosphorylation a plausible but unproven contributor to focal adhesion dysfunction in CEAS.

In the CEAS–CD shared networks, *CDH1* (E-cadherin) was the top-ranked hub gene in the proteomics network, forming a densely connected module with junction proteins (*CTNND1*, *VCL*, *TJP3*, *CGN*, and *DSP*). Disruption of E-cadherin-mediated adhesion has been linked to epithelial barrier dysfunction in IBD [15,16,17]. *VIM* (vimentin), a top-ranked hub gene in the shared phosphoproteomics network, is a NOD2-interacting protein involved in NF-κB signaling and autophagy [18,19]. Vimentin-positive stromal cells expressing COX-2 have been shown to restrain innate immune responses in colitis through PGE2 production [20], which is of potential relevance to CEAS given the central role of PGE2 transport dysfunction mediated by OATP2A1.

To identify the features most robust to statistical correction, we examined the proteins retaining significance after FDR correction in the CEAS vs. Control comparison. Within this subset, several proteins involved in brush-border structure and epithelial homeostasis, including *CDHR2*, *MUC3A*, *MUC13*, *ALPI*, and *NDRG1*, were significantly downregulated [21], while actin cytoskeletal regulators (*TPM1*, *TPM3*, *FLNB*, *ZYX*, *SORBS1*, and *DES*) remained enriched. Loss of brush-border integrity and actin cytoskeletal reorganization therefore emerge as the most statistically robust molecular features identified in CEAS.

Taken together, our findings suggest that CEAS is characterized by coordinated disruption of epithelial surface architecture, encompassing impairment of the brush border and apical membrane, actin cytoskeletal reorganization, and alterations in cell–cell junctions and cell–matrix adhesion systems. Focal adhesion-related alterations emerged consistently across multiple analytical approaches; however, because the principal hub proteins did not remain significant after FDR correction, their mechanistic relevance to CEAS requires further validation [22,23,24,25]. Among the 10 CEAS-specific proteins showing concurrent expression and phosphorylation changes, several are involved in cytoskeletal dynamics and focal adhesion (*TLN1*, *FLNA*, and *SORBS1*), whereas the seven shared proteins (including *CGN*, *DSP*, and *TJP3*) are predominantly associated with intercellular junctions.

This study has several limitations. The small sample size, inherent to this rare disease, limited statistical power; accordingly, the findings should be considered exploratory and hypothesis-generating. The use of archival FFPE tissue may have introduced protein degradation artifacts, and the cross-sectional design precludes causal inference. Finally, the major PPI-derived hub proteins did not remain significant after FDR correction and require further validation.

In conclusion, this exploratory study suggests that coordinated disruption of epithelial surface architecture, including impairment of the intestinal brush border and mucosal barrier as well as actin cytoskeletal reorganization, represents a central molecular feature of CEAS, whereas adaptive immune activation more strongly characterizes CD. *POSTN*, *CDH1*, *TLN1*, *VIM*, and focal adhesion-related pathways are presented as candidates for future mechanistic investigation.

## Figures and Tables

**Table 1:** Summary of differentially expressed proteins and differentially phosphorylated proteins.

Dataset	Comparison	Treatment	Control	Upregulated	Downregulated
Proteomics	1	CEAS	Normal	484	416
	2	CD	Normal	106	171
	3	CEAS	CD	82	93
Phosphoproteomics	1	CEAS	Normal	100 *	55 *
	2	CD	Normal	51 *	54 *
	3	CEAS	CD	61 *	23 *

CEAS, *SLCO2A1*-related chronic enteropathy; CD, Crohn’s disease. * Phosphoproteomics values represent differentially phosphorylated sites. After gene-level deduplication, 121, 80, and 64 DPPs were identified for the three comparisons, respectively.

**Table 2:** Summary of gene clusters and functional enrichment from hierarchical clustering analysis.

Cluster	Genes	Top Enriched Function	*p*-Value	Key Genes
1	18	H_2_O_2_ catabolic process	5.20 × 10^−11^	*HBB*, *HBA1*, *HBD*, *MPO*
2	23	Ig-mediated immune response	1.65 × 10^−9^	*IGHA1*, *IGKC*, *IGHG1*
3	10	Platelet aggregation	6.27 × 10^−6^	*MYL9*, *MYH11*, *VCL*
4	25	Glycolytic process	3.17 × 10^−6^	*GAPDH*, *ENO1*, *ALDOA*, *PKM*
5	24	Epithelial cell apoptotic process	1.61 × 10^−8^	*FGA*, *FGB*, *FGG*, *KRT8*

**Table 3:** Summary of significantly enriched gene sets identified by GSEA across proteomic and phosphoproteomic comparisons.

Dataset	Comparison	Database	Total	Up	Down	Top Pathway (NES)
Proteomics	CEAS vs. Normal	GO BP	145	79	66	Digestion (−2.94)
Proteomics	CEAS vs. Normal	KEGG	20	15	5	Focal adhesion (2.22)
Proteomics	CD vs. Normal	GO BP	16	11	5	Adaptive immune (3.24)
Proteomics	CD vs. Normal	KEGG	2	1	1	Cytoskeleton (1.65)
Proteomics	CEAS vs. CD	GO BP	27	5	22	Adaptive immune (−2.78)
Proteomics	CEAS vs. CD	KEGG	0	—	—	—
Phosphoproteomics	CEAS vs. Normal	GO BP	0	—	—	—
Phosphoproteomics	CEAS vs. Normal	KEGG	0	—	—	—
Phosphoproteomics	CD vs. Normal	GO BP	4	1	3	Cellular localization (−1.98)
Phosphoproteomics	CD vs. Normal	KEGG	0	—	—	—
Phosphoproteomics	CEAS vs. CD	GO BP	9	0	9	Nucleic acid metabolic process (−2.15)
Phosphoproteomics	CEAS vs. CD	KEGG	0	—	—	—

Significance criteria: |NES| > 1, nominal *p* < 0.05, and FDR < 0.25. NES, normalized enrichment score; GO BP, Gene Ontology Biological Process; —, not applicable.

**Table 4:** Top 10 hub genes identified by MCC algorithm in PPI networks.

(**a**) **CEAS-Specific DEPs (648 Nodes)**							
**Tied Rank**	**Gene**	**MCC Score**	**Degree**	**Ratio**	**Nominal *p***	***q* (BH)**	**FDR Status**
1	*COL1A1*	2.72 × 10^9^	46	2.76	0.044	0.164	No
2	*COL1A2*	2.72 × 10^9^	48	2.27	0.025	0.124	No
3	*COL3A1*	2.71 × 10^9^	40	1.94	0.011	0.096	Trend
4	*COL4A1*	2.70 × 10^9^	28	2.25	0.0017	0.051	Trend
5	*COL5A1*	2.70 × 10^9^	33	2.54	0.037	0.148	No
6	*FN1*	2.69 × 10^9^	94	1.67	0.021	0.117	No
7	*ELN*	2.58 × 10^9^	33	0.48	0.038	0.151	No
8	*THBS2*	2.57 × 10^9^	28	3.05	0.043	0.162	No
9	*POSTN*	2.53 × 10^9^	39	3.47	0.028	0.130	No
10	*THBS1*	1.59 × 10^9^	37	0.40	0.0068	0.081	Trend
(**b**) **CEAS–CD Shared DEPs (134 Nodes)**							
**Tied Rank**	**Gene**	**MCC Score**	**Degree**	**Ratio**	**Nominal *p***	***q* (BH)**	**FDR Status**
1	*CDH1*	569	28	0.35	0.0068	0.081	Trend
2	*CTNND1*	421	14	0.63	0.020	0.115	No
3	*VCL*	387	20	1.72	0.033	0.142	No
4	*TJP3*	360	9	0.44	0.0057	0.078	Trend
5	*PLEKHA7*	288	8	0.49	0.031	0.137	No
6	*CGN*	240	8	0.34	0.021	0.117	No
7	*DSP*	224	11	0.44	0.0064	0.081	Trend
8	*KRT19*	164	13	0.34	0.029	0.131	No
9	*KRT8*	132	10	0.35	0.0051	0.075	Trend
10	*F11R*	109	9	0.41	0.0021	0.053	Trend
(**c**) **CEAS-Specific DPPs (43 Nodes)**							
**Tied Rank**	**Gene**	**MCC Score**	**Degree**	**Ratio**	**Nominal *p***	***q* (BH)**	**FDR Status**
1	*TPM1*	64	10	2.50	0.031	0.231	No
2	*CALD1*	58	10	2.55	0.045	0.261	No
3	*FLNA*	49	9	3.34	0.042	0.257	No
4	*TLN1*	39	10	2.27	0.029	0.223	No
5	*TAGLN*	38	7	5.73	0.020	0.199	No
6	*CNN1*	29	7	3.89	0.0025	0.109	No
7	*SORBS1*	26	5	2.98	0.0067	0.148	No
8	*SORBS3*	24	4	2.69	0.019	0.199	No
9	*DBN1*	13	5	1.83	0.019	0.199	No
10	*SYNM*	10	5	2.15	0.0031	0.111	No
10	*CAV1*	10	6	2.56	0.011	0.183	No
(**d**) **CEAS–CD Shared DPPs (27 Nodes)**							
**Tied Rank**	**Gene**	**MCC Score**	**Degree**	**Ratio**	**Nominal *p***	***q* (BH)**	**FDR Status**
1	*CTTN*	4	4	0.45	0.036	0.239	No
2	*VIM*	3	3	2.62	0.00085	0.071	Trend
2	*PDLIM2*	3	3	0.32	6.1 × 10^−5^	0.028	Yes
2	*DPYSL3*	3	3	1.89	0.021	0.199	No
5	*PDLIM4*	2	2	6.21	0.0058	0.141	No
5	*KLC4*	2	2	0.48	0.012	0.183	No
5	*DPYSL2*	2	2	1.51	0.044	0.258	No
5	*EML4*	2	2	0.20	0.0015	0.083	Trend
5	*PALM2AKAP2*	2	2	—	—	—	NQ
5	*SRRM2*	2	2	1.89	0.048	0.272	No

PPI, protein–protein interaction; MCC, Maximal Clique Centrality; DEPs, differentially expressed proteins; DPPs, differentially phosphorylated proteins. Hub genes were identified using the cytoHubba plugin in Cytoscape. Degree indicates the number of direct interactions in the STRING network (confidence ≥ 0.400). *q* (BH), Benjamini–Hochberg-adjusted *q*. FDR status: Yes, *q* < 0.05; Trend, *q* < 0.10; No, *q* ≥ 0.10; NQ, not quantified.

## Data Availability

The data presented in this study are available on request from the corresponding author.

## Supplementary Materials

The following supporting information can be downloaded at: https://www.mdpi.com/article/10.3390/biomedicines14071412/s1, Figure S1: Normalized enrichment score (NES) barplots summarizing all significantly enriched gene sets identified by GSEA across proteomic comparisons; Figure S2: Molecular docking of PGE_2_ with four candidate hub proteins using AutoDock 4.2; Figure S3: Quality-control metrics for the proteomic and phosphoproteomic datasets; Table S1: Molecular docking results of PGE_2_ with four hub proteins; Table S2: Benjamini–Hochberg FDR sensitivity analysis of differential expression; Table S3: Clinical and sample characteristics of the study cohort.
