# Supplementary material for: Integrative Proteomics and Phosphoproteomics Profiling of Chronic Enteropathy Associated with SLCO2A1 Gene Reveals Mucosal Barrier Impairment and Focal Adhesion Pathway Alterations
Source: Biomedicines. 2026 Jun 23;14(7):1412. doi: 10.3390/biomedicines14071412 (PMC13404605; doi:10.3390/biomedicines14071412)
Supplement: Supplementary file 1 [file biomedicines-14-01412-s001.zip › biomedicines-4298050-supplementary.pdf]

Supplementary Figure S1. Normalized enrichment score (NES) barplots summarizing all significantly enriched gene sets identified by GSEA across proteomic comparisons. (a, b) CEAS vs. Normal: GO biological process and KEGG pathways. (c, d) CD vs. Normal: GO biological process and KEGG pathways. (e, f) CEAS vs. CD: GO biological process and KEGG pathways. Red bars indicate positive NES (enriched among upregulated proteins) and blue bars indicate negative NES (enriched among downregulated proteins). Significance criteria: |NES| > 1, nominal P < 0.05, and FDR < 0.25. No significant KEGG pathways were identified in the CEAS vs. CD comparison.

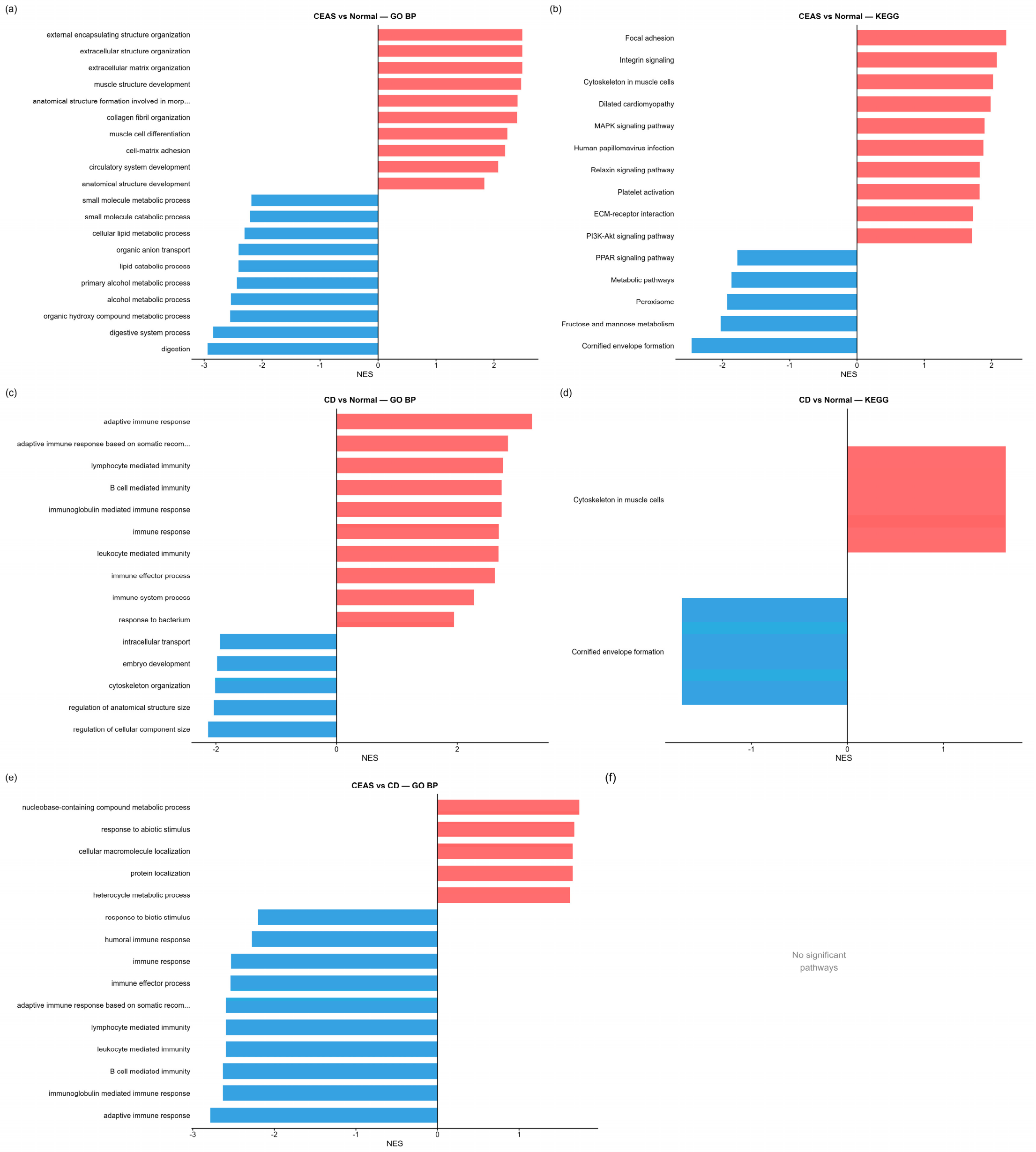

Supplementary Figure S2. Molecular docking of PGE_2 with four candidate hub proteins using AutoDock 4.2. (a) CDH1. (b) POSTN. (c) TLN1. (d) VIM. Each panel shows the best-ranked docking pose of PGE_2 on the protein surface.

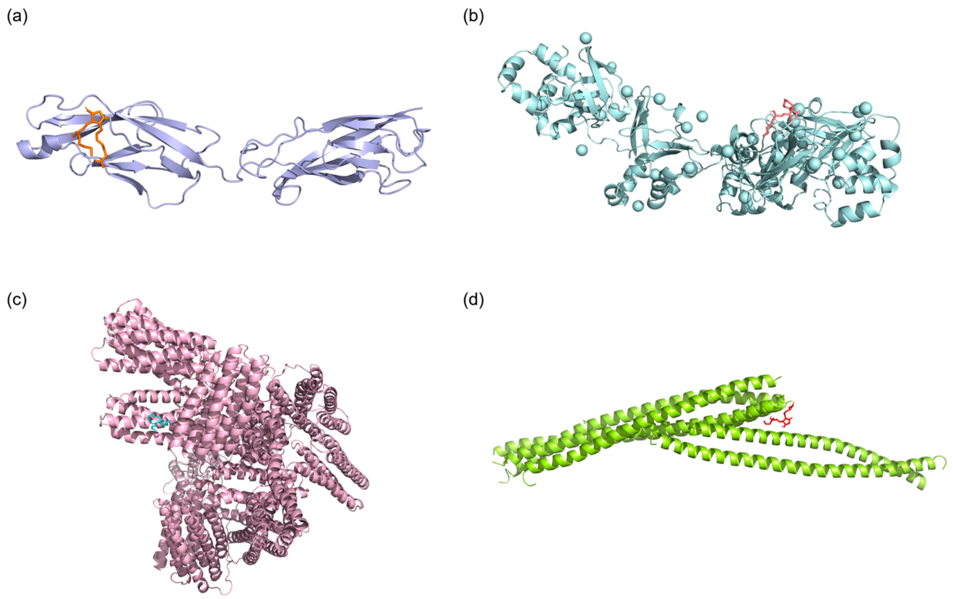

Supplementary Figure S3. Quality-control metrics for the proteomic and phosphoproteomic datasets. (a) Principal component analysis (PCA) of the proteomic data, showing separation of CEAS, CD, and control groups. (b) Pairwise Pearson correlation of protein quantification across all nine samples (values indicate R between sample pairs). (c) Distribution of protein quantification intensities before and after normalization (proteomic dataset); each box represents one sample, with comparable medians after normalization indicating consistent sample processing. (d) Mass error distribution of the phosphoproteomic dataset, with the majority of peptide-spectrum matches within ±10 ppm.

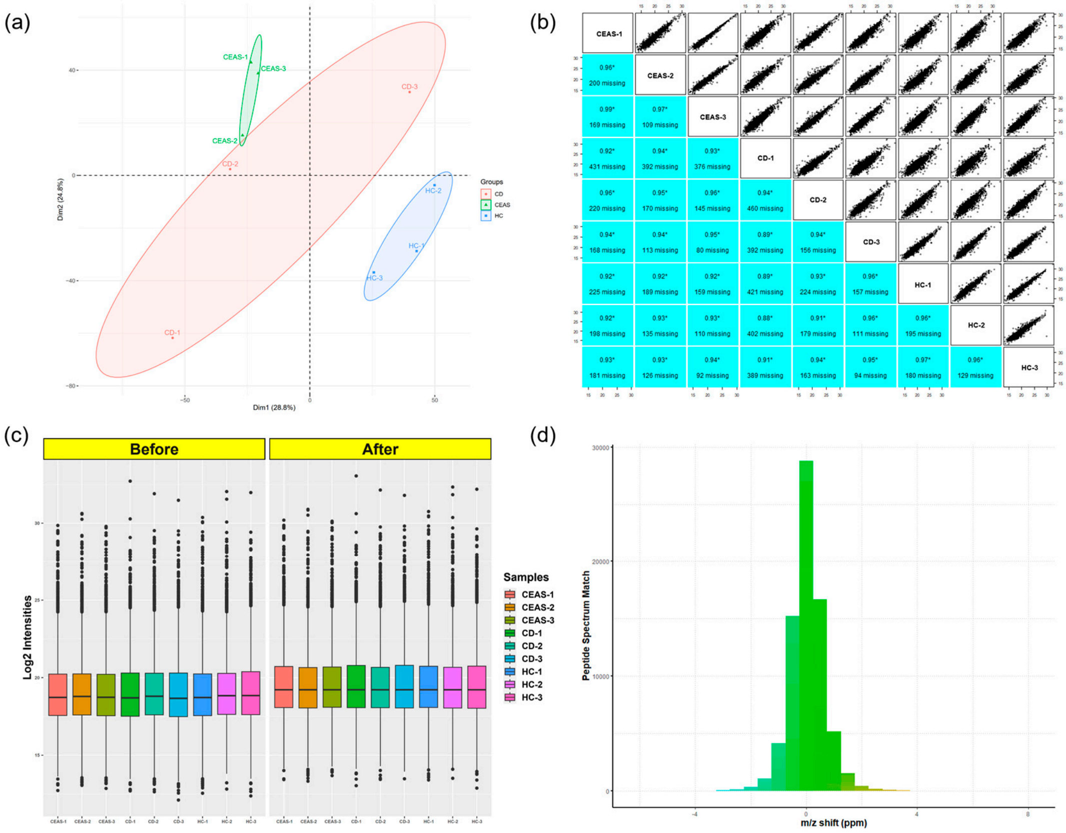

Supplementary Table S1. Molecular docking results of PGE_2 with four hub proteins.

Target protein	Best ΔG (kcal/mol)	No. of clusters	Largest cluster size	Total runs
CDH1	-2.95	46	2	50
POSTN	-2.62	49	2	50
TLN1	-2.51	48	2	50
VIM	-2.04	49	2	50

Supplementary Table S2. Benjamini–Hochberg FDR sensitivity analysis of differential expression.

Dataset	Comparison	Total tested	Nominal (P≤0.05 & FC)	BH q<0.05 & FC	BH q<0.10 & FC	Min q
Proteomics	CEAS vs Control	4753	900	128	437	0.0042
Proteomics	CD vs Control	4753	277	0	0	0.139
Proteomics	CEAS vs CD	4753	175	0	1	0.077
Phosphoproteomics	CEAS vs Control	921	155	2	18	0.028
Phosphoproteomics	CD vs Control	921	105	0	1	0.087
Phosphoproteomics	CEAS vs CD	921	84	0	0	0.136

FC, fold change (≥1.5 or ≤0.67); nominal P, unadjusted Student's t-test; q, Benjamini–Hochberg-adjusted P value.

Supplementary Table S3. Clinical and sample characteristics of the study cohort.

Variable	CEAS-1	CEAS-2	CEAS-3	CD-1	CD-2	CD-3	HC-1	HC-2	HC-3
Age (years)	37	41	47	49	49	30	35	48	70
Sex	M	M	M	M	M	M	M	M	M
Ethnicity	Han	Han	Han	Han	Han	Han	Han	Han	Han
BMI (kg/m^2)	19.0	14.7	16.8	15.6	19.9	26.1	31.6	27.7	23.9
SLCO2A1 variant	Confirmed, variant ND	Confirmed; parents (−)	Confirmed; mother & son carriers	NA	NA	NA	NA	NA	NA
PHO manifestation	Yes	No	Yes	NA	NA	NA	NA	NA	NA
Age at onset (years)	22	28	26	39	36	16	NA	NA	NA
Disease duration (years)	15	13	21	10	13	14	NA	NA	NA
Lesion location	Ileum	Diffuse small bowel	Terminal ileum	Distal jejunum, ileum, ileocecum, cecum, proximal ascending colon	Ileum, right colon	Ileocecum, ascending colon	Small bowel	Small bowel	Ileum
Medication at sampling	Etoricoxib 60 mg qd, mesalazine	Etoricoxib 60 mg qd	None	Azathioprine 50 mg qd, mesalazine	Thalidomide 50 mg qd, mesalazine	Ustekinumab	NA	NA	NA
CRP (mg/L)	7.84	34.2	2.67	2.36	83.79	108.8	238.3	62.86	244.64
Hemoglobin	75	75	94	149	101	128	151	72	142

(g/L)									
Sampling method	Surgical resection	Endoscopic biopsy	Endoscopic biopsy	Surgical resection	Surgical resection	Surgical resection	Surgical resection	Surgical resection	Surgical resection
Sampling site	Ileal stricture	Terminal ileum	Terminal ileum	Terminal ileum	Terminal ileum	Terminal ileum	Terminal ileum	Terminal ileum	Terminal ileum
FFPE storage time (years)	2	2	3	1.5	2	2	2	2	2
